# PRO: Biomarker surveillance for invasive fungal infections without antifungal prophylaxis could safely reduce antifungal use in acute leukaemia

**DOI:** 10.1093/jacamr/dlac074

**Published:** 2022-07-22

**Authors:** Thomas Taynton, Gavin Barlow, David Allsup

**Affiliations:** Hull University Teaching Hospitals NHS Trust, Castle Hill Hospital, Castle Road, Cottingham, Hull, HU16 5JQ, UK; Centre for Atherothrombosis and Metabolic Disease, Hull York Medical School, University of Hull, Hull, HU6 7RX, UK; Hull University Teaching Hospitals NHS Trust, Castle Hill Hospital, Castle Road, Cottingham, Hull, HU16 5JQ, UK; Hull York Medical School, University of York, Heslington, York, YO10 5DD, UK; Hull University Teaching Hospitals NHS Trust, Castle Hill Hospital, Castle Road, Cottingham, Hull, HU16 5JQ, UK; Centre for Atherothrombosis and Metabolic Disease, Hull York Medical School, University of Hull, Hull, HU6 7RX, UK

## Abstract

Mould-active antifungal prophylaxis is frequently used to prevent invasive fungal infection in patients with acute leukaemia being treated with intensive chemotherapy. Invasive fungal infections are difficult to diagnose, and despite the use of prophylaxis a high proportion of patients still receive therapeutic antifungals. Antifungal medications have important interactions, can cause serious adverse events, and may drive the proliferation of antifungal resistance. The use of two biomarkers, such as galactomannan in combination with the less-specific β-d-glucan, can mitigate the risk of not detecting non-*Aspergillus* species, as well as improving pooled sensitivity and specificity. We argue that regular biomarkers could be used safely as part of an antifungal stewardship strategy to reduce antifungal use, by both screening for infection in patients not on prophylaxis and ruling out infection in patients treated empirically.

The diagnosis of an invasive fungal infection (IFI) in patients undergoing intensive chemotherapy for acute leukaemia is undoubtedly difficult.^1^ Proven infection requires sterile material from the affected site^2^ (most commonly the lung) and the use of bronchoscopy and lavage (BAL) to obtain specimens carries an increased risk of adverse events in patients with pancytopenia. Additionally, such investigations may not be deliverable in a critically ill, immunocompromised patient. Less invasive diagnostic methods, such as blood biomarkers, are highly desirable and increasingly used, but a controversial question is whether biomarker surveillance during periods of risk can replace mould-active antifungal prophylaxis. We believe it can.

A landmark study in 2007 found that patients with acute myeloid leukaemia or myelodysplastic syndrome who received antifungal prophylaxis using posaconazole, compared with fluconazole or itraconazole, had significantly lower incidences of invasive aspergillosis (2% versus 8%) and 100 day all-cause mortality (14% versus 21%).^3^ The IDSA,^4^ European Conference of Infections in Leukaemia (ECIL),^5^ German Society of Haematology and Medical Oncology (DGHO)^6^ and ESCMID^7^ all recommend using posaconazole in high-risk patients predicted to have a prolonged episode of neutropenia related to intensive chemotherapy. Meta-analyses have also shown reductions in IFI and IFI-related mortality with the use of mould-active prophylaxis versus either fluconazole or placebo, but have not shown a reduction in overall mortality,^8^ including a posaconazole-specific study from 2020.^9^

A fundamental problem with prophylaxis is that despite all patients receiving an antifungal during high-risk periods, a high proportion of patients still receive additional therapeutic antifungals: 27% in the posaconazole group in the Cornely *et al.* trial^3^ with only 5% deemed to have had probable or proven IFI. In some reports, exposure to therapeutic antifungals is nearly 50% in real-world populations of intensive chemotherapy-treated patients.^10^ Even in high-risk patients, continuing such an approach in the era of emerging antimicrobial resistance is highly undesirable, particularly as challenging to diagnose and treat breakthrough infections still occur despite prophylaxis.^11^

Resistance to azoles is increasing for both *Candida* and *Aspergillus* species.^12^ MDR *Candida auris* is an exemplar of a pathogenic fungus that can become endemic both in community and healthcare environments, results in considerable infection control challenges, and causes life-threatening infections in vulnerable populations. We know that the use of antifungals promotes colonization with resistant fungi,^13^ and our knowledge of the unrelenting march of antibiotic resistance in Gram-negative bacteria and *Mycobacterium tuberculosis* should forewarn us that we need to optimize antifungal use now. Global and national antimicrobial stewardship programmes are increasingly acknowledging the importance of including antifungal agents in their strategies.^10,14^

Antifungal drugs also carry a risk of adverse events, such as gastrointestinal disturbances and hepatic and cardiac toxicity.^15^ Posaconazole specifically requires an acidic environment for gastrointestinal absorption, which can be problematic as proton-pump inhibitors are commonly taken by patients with acute leukaemia.^16^ Interactions can also occur due to inhibition of cytochrome P450 3A4, notably with an increased incidence of vincristine toxicity in patients taking posaconazole.^17^ To avoid such interactions alternative antifungal prophylaxis, such as weekly liposomal amphotericin, with less convincing efficacy data, is required. In our experience, patients also prefer to take fewer medications.

Preventing and treating IFIs is expensive. In our own hospital the use of posaconazole and liposomal amphotericin accounts for two-thirds of the hospital’s total antifungal costs. A German study found the incremental cost of treatment of an IFI was €21 063, 36% of which was the antifungal.^18^ A number of new antifungals are in development, but these will be expensive. A non-prophylaxis approach, however, must undoubtedly ensure that drug-related savings are not lost by an increase in infections or poorer outcomes.

A biomarker-based surveillance approach is mentioned as an alternative in the ESCMID guideline^7^ and is used in some centres that have not experienced an increased incidence of IFIs. A surveillance approach could involve regular, twice-weekly biomarker monitoring with clinical assessment and further biomarker-driven investigation for IFI prior to the onset of clinical symptoms. Given a relatively high negative predictive value, negative surveillance biomarkers could provide reassurance to clinicians that empirical antifungal therapy is not required in the presence of prolonged fever and encourage cessation of empirical therapy when the risk of IFI has been assessed as low. There is a lack of high-quality evidence to allow robust comparisons of antifungal prophylaxis versus diagnostic approaches without prophylaxis, but even when used with prophylaxis diagnostic strategies in the empirical setting appear to reduce antifungal prescribing safely. A previous prospective observational study used thrice-weekly galactomannan (GM) testing combined with CT scanning (± BAL) in patients treated with fluconazole prophylaxis. Mould-active antifungal treatment was only advised if there was both mycological and radiological evidence of IFI; this approach found substantial reductions in antifungal use during episodes of neutropenic fever (from 35% to 7.7%).^19^ The randomized trial by Morrissey *et al.*,^20^ which compared a standard culture and histology diagnostic strategy versus a GM plus *Aspergillus* PCR approach, supports these findings with an associated reduction in empirical antifungal therapy from 32% to 15%. Importantly, the increase in probable fungal infection in the GM/PCR group was thought to be due to the diagnostic inferiority of the traditional approach and was not associated with an increase in all cause of IFI-related mortality. A multicentre Spanish study compared surveillance with GM and *Aspergillus* PCR to GM alone in leukaemia patients not receiving any antimould prophylaxis; rates of IFI in the GM-PCR group were lower than GM alone (4.2% versus 13.1%). Whilst not directly comparable, these latter results are similar to IFI rates of patients on prophylaxis in other trials.^21^ In the UK, a relatively large observational study investigated a diagnostic-driven approach where patients, mostly treated with itraconazole prophylaxis, had regular biomarker monitoring that informed management in the empirical setting. This study appeared to produce favourable results with a reduction in unnecessary antifungal use without excess mortality.^22^

One of the concerns with any diagnostic approach that specifically targets *Aspergillus* species, using for example GM or *Aspergillus* PCR alone or in combination without antifungal prophylaxis, is that other IFIs may not be detected. Although these occur less commonly than *Aspergillus* infections when prophylaxis is administered (e.g. 2% in the trial by Cornely *et al.*^3^), this risk is potentially mitigated by the use of a second non-specific IFI biomarker such as β-d-glucan that can also detect *Candida*, *Pneumocystis* and some other non-*Aspergillus* fungal species.^23^ There have also been concerns about the performance of IFI biomarkers during antifungal prophylaxis.^24^ Indeed, the decrease in the rate of diagnosis of IFI without an associated reduction in overall survival in many of the meta-analyses of prophylaxis could be due to this reduced performance of biomarkers or other reasons, such as mortality being predominantly driven by the underlying leukaemia rather than IFI.

A biomarker-based diagnostic strategy may also be cost-effective. An economic comparison, based on UK costings, compared a standard strategy of empirical treatment for IFI in patients with 72–96 h of neutropenic fever to only initiating antifungal therapy if the patient screened positive on biomarkers (GM or *Aspergillus* PCR) or an abnormal CT scan. The diagnostic-driven strategy was cost-saving, although this study was model-based; neither arm received antimould prophylaxis.^25^ A similar Australian economic comparison, where patients had a mixture of different antifungal prophylaxis, found that a diagnostic strategy was cost-effective if there was a survival benefit.^26^ What we do know is that reducing the use of antifungals empirically when there is no evidence of IFI appears to reduce costs without adversely affecting mortality.^27^

Biomarkers have been criticized for having poor individual sensitivity and specificity for diagnosing IFI, and higher than desirable false positive and negative results. Variation in the performance characteristics reported between studies is likely to be due to multiple factors including, for example, the assay used, positivity cut-offs adopted and the patient population.^23,28^ There is evidence that combining assays can improve pooled sensitivity and specificity, but the optimal approach is yet to be identified or adequately tested.^29^ A large trial comparing antimould prophylaxis directly to surveillance biomarkers without prophylaxis, such as the BioDriveAFS trial,^30^ which is due to start in the UK in 2022, is justified and overdue.

We do not advocate a one-size-fits-all approach and recognize we are on an evolving pathway towards personalized IFI prevention, where the highest-risk patients, identified before and during periods of risk by emerging technologies, are targeted for antifungal prophylaxis while most undergo biomarker surveillance. Even without such scientific advances, in selected high-risk patients there may still be a role for antifungal prophylaxis when external factors, such as construction work, are present.^31^ Another limitation in the appreciation of IFI as a clinical problem is the lack of mandatory reporting of IFI in the UK with rates of infection based on estimates. This acts as a barrier to haematology units knowing what their level of risk is and how they benchmark against other comparable units.^32^ Identifying patient groups and units with high rates of IFI, and more importantly understanding why this is the case, is a research gap that needs to be addressed.

There is mounting evidence to demonstrate that IFI biomarkers can be used safely to guide the prescription of antifungals in certain patient groups in both the surveillance and empirical settings. Undoubtedly this area needs to be investigated further within robustly designed and delivered clinical trials. Based on the evidence to date, a diagnostic-driven approach appears to reduce antifungal use safely in the management of patients with acute leukaemias.^33^ In the future, a personalized strategy will be the optimal approach, and this is what the IFI research community should be working towards.
